# A Smartphone Magnetometer-Based Diagnostic Test for Automatic Contact Tracing in Infectious Disease Epidemics

**DOI:** 10.1109/ACCESS.2019.2895075

**Published:** 2019-01-25

**Authors:** Seungyeon Jeong, Seungho Kuk, Hyogon Kim

**Affiliations:** Department of Computer Science and EngineeringKorea University 34973 Seoul02841South Korea

**Keywords:** Mobile sensing, human contact tracing, smartphone magnetometer, infectious disease epidemic, diagnostic test

## Abstract

Smartphone magnetometer readings exhibit high linear correlation when two phones coexist within a short distance. Thus, the detected coexistence can serve as a proxy for close human contact events, and one can conceive using it as a possible automatic tool to modernize the contact tracing in infectious disease epidemics. This paper investigates how good a diagnostic test it would be, by evaluating the discriminative and predictive power of the smartphone magnetometer-based contact detection in multiple measures. Based on the sensitivity, specificity, likelihood ratios, and diagnostic odds ratios, we find that the decision made by the smartphone magnetometer-based test can be accurate in telling contacts from no contacts. Furthermore, through the evaluation process, we determine the appropriate range of compared trace segment sizes and the correlation cutoff values that we should use in such diagnostic tests.

## Graphical abstract

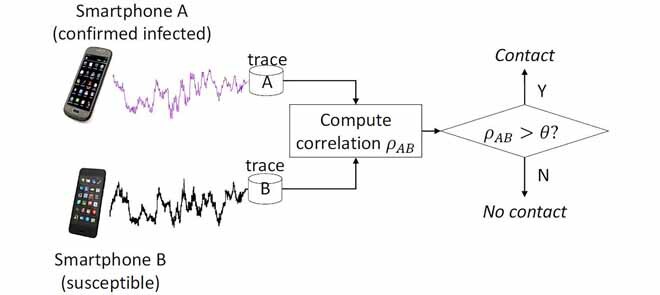

## I. Introduction

Witnessing an alarmingly large number of novel pandemics in this century such as SARS, Swine Flue, MERS, Ebola, and Zika, there has been growing concerns on the “next big one” [1]. Many worry that we deal with them using strategies established over a century ago and technology that has been around for decades, with little innovation generated [2]. Consequently, there are calls for technology-based preparedness [3], especially in the areas of infection prevention, case finding, case investigation, and contact tracing [4]. Among others, the information technology (IT) sector should respond to the calls, and continuously expand and finesse the arsenal of technologies in each of these areas. In this paper, we tackle one of the areas that need the technological revamping: *contact tracing*.

On the brink of an infectious disease epidemic, the most urgent task is to trace those who possibly made contacts with the infected person(s), in order to cut the chain of infection and prevent it from growing into a wider epidemic. But the traditional contact tracing technique has been predominantly analog. Namely, contact graphs are constructed through interviews with confirmed cases, by asking who they met and where they visited. This is a hugely costly and time consuming task. Worse yet, there is the issue of recall [5]. Meanwhile, recent outbreaks have been fundamentally different from those of the past – highly mobile populations [6] and the spread into densely populated cities [2] – which exacerbate the problem with the traditional contact tracing approach.

When there are many potential contacts that an infected person cannot identify or recollect as in our typical urban life, a potent tool we can marshal is the mobile devices such as smartphones. The mobile-based epidemic monitoring is nothing but a logical next step because only the mobile devices that move with people can keep up with the contacts they make. Indeed, there have been increasing number of proposals for smartphone-based contact tracing. The employed technologies range from similar Global Positioning System (GPS) positions [7], similar Wi-Fi fingerprints [8], Bluetooth peer discovery [9], and identical cells in mobile communication [10]. Unfortunately, they either provide position information too coarse to be used for infectious contact detection [11] (GPS, cellular/Wi-Fi fingerprinting), require the infrastructure nearby (cellular/Wi-Fi), cannot be used indoors (GPS), consumes too much power for extended monitoring use (GPS) [12], or could compromise privacy by exposing the identity of the device and eventually its owner (Bluetooth beacons).

However, some recent works including our earlier pilot study [11], [13] present a new possibility by demonstrating that a magnetometer traces-based approach can detect close contacts. They exploit the fact that the magnetic field strength is rich in spatial features (*e.g.* 1 m^−1^ to 0.01 m^−1^) [14] due to various distortions by ferromagnetic materials used in buildings such as reinforced concrete and metal doors. The magnetometer-based approach overcomes most of the aforementioned issues. First, thanks to the omnipresent geomagnetic field, the similarity comparison of the two magnetometer traces works both indoors and outdoors, and does not need any infrastructure support. Second, it offers better privacy protection by not revealing any identity of the device or the location of the trace generation. Third, it detects the coexistence only in close proximity. The low-power smartphone magnetometers can only be affected by ferromagnetic structures within a few meters [15], [16]. Only the co-existing smartphones within this distance can bear sufficient similarity in their magnetometer readings [11], [17]. This last characteristics is especially important as many infectious disease transmissions occur in close distances. Public health policies for tracing close contacts or infection control guidance often use a distance of up to 2 meters or 6 feet [18].

**FIGURE 1.**
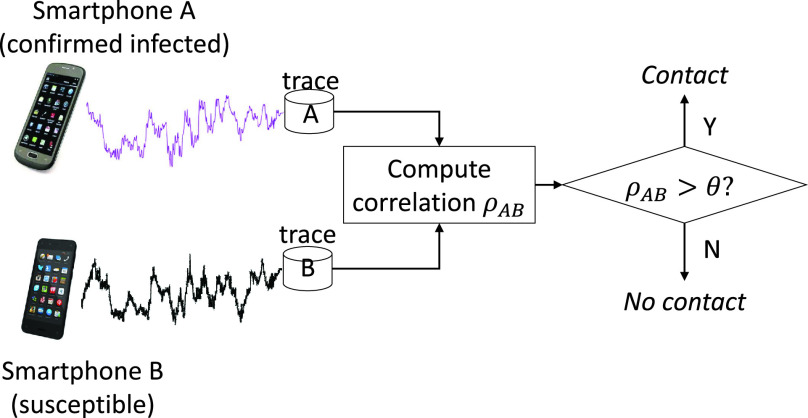
A possible diagnostic test by exploiting the magnetometer traces. It can pre-screen if the susceptible person “B” had a (possibly unknown) contact with person “A” who has already been confirmed infected. The disease control authority can release the trace of the infected without revealing his or her identity for the checking use.

When the disease control authority performs an epidemiological investigation, they can use the smartphone magnetometer traces of the person confirmed infected and of the one suspected of a contact with the infected, in a system depicted in Fig. 1. When people make contacts, they are recorded in their individual phones in the form of similar magnetometer readings. When they want to check if they could have met an infected person, they can ask the system to compare their traces with that of the infected person. Since it is a pairwise comparison, it works for the case many people gather at a location. Each individual pair from the gathering can be checked using the pairwise comparison method depicted in Fig. 1. In this paper, we assume that it is an emergency situation, and people are cooperating with the disease control authority by downloading an application that records the magnetometer readings and submits it through the phone’s cellular connection if necessary. Indeed, there are recent efforts that seek such public participation to prepare for the next pandemic outbreak [19]. In this effort by British Broadcasting Corporation (BBC), people are encouraged to download an app and activate it for helping model the spreading dynamics in future pandemics. The app then meticulously record the trajectory of the smartphone holder, before it reports the trajectory information to a central server. Considering this precedent, our own system model in Fig. 1 is not excessively unrealistic. The rationale behind such cooperation from the public could be the fear from the lack of information [20]. Under the depicted scenario, not only the disease control authority but also each individual user can check herself whether or not there has been close contact with an infected person. Indeed, World Health Organization (WHO) strongly recommends that disease control authorities ensure at-risk populations have the information they need, thereby minimizing social and economic disruption [21].

**FIGURE 2.**
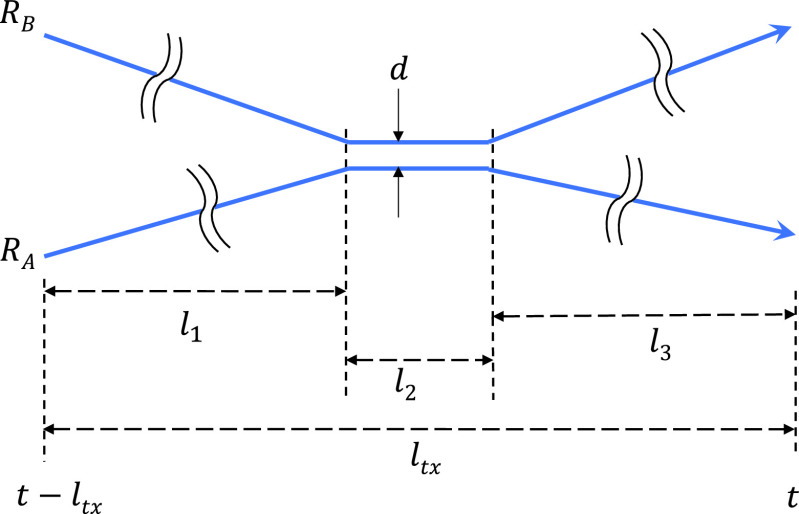
The actual contact time $l_{2}$ where the two smartphones are collocated within the disease transmissible critical distance $d$ can be relatively very small compared to $l_{tx}$, the entire time span of checked length in the magnetometer traces.

Although the existing magnetometer-based works have confirmed the feasibility of the idea, they are a far cry from a serious alternative to the traditional contact tracing method. First, many of the operating parameters are still to be determined. In particular, the exiting works [11], [13], [17] consider only two extreme and impractical cases: either continuous contact or no contact during the whole duration of comparison. However, when two people, possibly strangers, make a contact, its duration can be very small compared to the entire span of comparison (which can match the most active transmission period of the disease). In Fig. 2, if the infected person A was confirmed infected at time $t$ and the disease transmissible duration is $l_{tx}$, the similarity measure between the traces $R_{A}$ and $R_{B}$ can be computed low if the duration of contact $l_{2} \ll l_{tx}$. But this will be generally the condition that we will face in reality. Therefore, we need to define the window of comparison $T_{W}$ that we slide over the entire trace pair to find any contact (*i.e.*, the similarity measure over a threshold) to make it a valid test method.

Second, when new diagnostic tests are introduced, it is necessary to evaluate the comparative diagnostic accuracy and feasibility of this new test in comparison to the existing tests or the gold standard [22]. This ability and diagnostic accuracy can be quantified by calculating various measures such as sensitivity and specificity, positive and negative likelihood ratios, diagnostic odds ratio, *etc*. In this paper, we address these issues by defining the desirable length of $T_{W}$, the decision threshold $\theta _{c}$, and by evaluating the quality of the contact diagnosis under these parameter values.

As to the nature of the technology we propose in this paper, one can argue that it is only supplementary. In that we believe that the final confirmation about the infection event should be always made by human experts, it is true to a certain degree. It can be used to quickly identify possible contacts with relatively high accuracy, so that the human experts can focus on the most likely ones that have been identified by technology. However, at the same time, the technology covers areas that the traditional method could not. Without the technical support, it may be not only costly and time-consuming but impossible in many contact events. First, The authority may not be able to catch up with the speed of spreading when the epidemic is full-blown. Second, in many urbanized societies of today, we do not even know or remember those who happen to sit next to us in the bus or train or in a restaurant. In large-scale epidemics, the technology can quickly pan out even such contacts that cannot be recovered from the memory of the infected person. In these second sense, the technology will be indispensible.

The rest of this paper is organized as follows. In Section II, we briefly summarize the related work that exploits the geomagnetic field strength to detect location and coexistence. In Section III, we first discuss how we measure the similarity of two traces that signals a possible contact. Then we identify the parameters that determine the performance of the magnetometer-based contact test, and discuss how we will measure it. In Section IV, we evaluate the performance of the test using a set of real-life smartphone magnetometer traces. Finally, we conclude the paper in Section V.

Before delving into the discussion, we list the acronyms used throughout the paper in Table 1.

**TABLE 1:** Acronyms Used in This Paper

Acronym	Unabbreviated form
AUC	Area under curve
BLE	Bluetooth Low Energy
CDR	Call detail records
DDTW	Derivative Dynamic Time Warping
DTW	Dynamic Time Warping
DOR	Diagnostic Odds Ratio
FN	False negative
FP	False positive
GPS	Global Positioning System
IT	Information technology
LR	Likelihood ratio
MERS	Middle East respiratory syndrome
NTP	Network Time Protocol
RSS	Received signal strength
RSSI	Received signal strength indicator
RFID	Radio-frequency identification
PAN	Personal area network
PHS	Portable hot spot
SARS	Severe acute respiratory syndrome
SPDT	Same-place-different-time (disease transmission)
SPST	Same-place-same-time (disease transmission)
TN	True negative
TP	True positive
UAV	Unmanned areal vehicle
WHO	World Health Organization

## II. Related Work

There is rich literature on co-presence detection or its use on epidemiology and social studies. In terms of the employed technology, existing works range from sensors to communications to social media. We summarize them below, with brief remarks on their relevance to our problem or the relation to our approach.

### A. GPS

Although the disease transmissibility check in contact tracing needs not necessarily absolute but relative coordinates (*i.e*., relative to the infected person), one may well consider using GPS trajectories to determine the distance of contact. For instance, Qi *et al*. use GPS to track and visualize space-time activities for a flu transmission study [23]. Unfortunately, GPS is a power-inefficient sensor. As we need to amplify the signal and achieve a high processing gain due to the small received power, a significant reduction in battery time is inevitable. For instance, it can drain a smartphone battery in much less than a typical charge interval even with minimal activity [24], [25]. In attempts to mitigate the problem, we could activate GPS only when user movement exceeds the accuracy bound, or turn it off indoors by detecting the condition through other means such as the received signal strength (RSS) fingerprints of cell towers. Even if the power issue is resolved, however, problems remain. First, the distance estimate between two GPS sensors may include a large error because each can have an average error over 10 meters, when a few meters matter in disease transmissibility check. Second, and more importantly, GPS is incapable of checking for possible infection events indoors.

### B. RFID and Sensor Network

Many studies have used radio frequency identification (RFID) or sensor network technologies to understand infection and to prevent it in hospitals [26] and in schools [27]. Isella *et al*. use active tags to track contacts that take place in a pediatric ward for analyzing the structure of the contact data, it identifies the central groups that need close attention to prevent nosocomial infection prevention. Salathe *et al*. use TelosB motes carried by students in a school to obtain close proximity interactions data and develop a more effective vaccination strategy. It finds the small world phenomenon, and suggests a vaccination strategy based on the structure that is more effective than random vaccination. It is also used in social studies [28] and for security based on proximity [29].

Shafagh and Hithnawi [29] use ambient radio signals to detect other nodes in close proximity, for authentication between IoT devices before they connect. Bolić *et al*. use an enhanced RFID tags to mutually detect proximity to track. When attached to people, it can be applied for tracking interactions at social events [28]. But the biggest drawback of these approaches is that today’s smartphones hardly support RFID or personal area network (PAN) technologies other than Bluetooth. Due to the lack of deployment base among general public, they do not serve our purpose of massive mutual contact monitoring between strangers.

### C. Social Media and Search Records

Recently, there have been efforts to introduce social media such as Twitter to epidemic monitoring, for early detection, management, and control of epidemic outbreaks [30]–[33]. In particular, participatory surveillance using social networks to collect symptom reports to detect infectious disease outbreaks has been tried. However, most studies limit their scope to common and seasonally recurring health events such as Influenza due to the noisy nature of Twitter [34]. Moreover, this post-symptomatic reporting can take long time because some diseases go through long incubation period (*e.g.* 3 weeks in Middle East Respiratory Syndrome (MERS) [35]). Moreover, subjective symptom reports do not provide information specific enough for disease control authorities to construct contact traces and obtain contact contexts. Also, it gives us only collective statistics at coarse granularities, while contact tracing requires information on person-to-person interactions. In the same vein, search-based global disease trend tracking services [36] are not directly helpful to contact tracing in emergency response.

### D. WI-FI

For its prevalence, Wi-Fi is extremely popular for indoor localization. For example, there is a recent work that leverages on participatory sensing [37]. Again, as in GPS, we could consider using Wi-Fi assisted location information to determine the distance of contact, although the disease transmissibility check in contact tracing needs not necessarily absolute but relative coordinates. However, there are not many works in co-locating two devices using the technology. Existing works based on Wi-Fi are mostly centered around proximity detection and its applications. But mutual proximity detection is not in the design of Wi-Fi, so it requires significant manipulation such as exploiting portable hot spot (PHS) mode [38]. Carreras *et al*. [38] use Wi-Fi to mutually discover smartphones in proximity and determine the distance using received signal strength indication (RSSI). In line-of-sight condition, they argue that 0.5 m resolution is achievable using the RSSI of the discovered smartphone and machine learning algorithms. As to the closeness estimation, most previous works rely on RSSI [39]–[41]. The applications include authentication [39], [40] and epidemic prediction [8]. In particular, Nguyen *et al*. [8] show that the co-presence in disease transmissible distance can be determined through RSSI signatures from public Wi-Fi access points. A drawback of using Wi-Fi is that access points may be unavailable or prove insufficient to fix positions with a consistent precision. Also, the technology is not stellar in energy efficiency, especially for long and continual monitoring.

### E. Bluetooth

For proximity detection, Bluetooth is a popular technology for its relatively high precision in short distances [9]. It has been mostly used for studies on social behavior and interaction such as duration and proximity [42], and those in mass gathering situations [43]. Liu *et al*. [9] show that Bluetooth can be used to detect face-to-face interaction within 1.5 m by mapping Bluetooth RSSI to distance. In this work, smartphones attempt to detect other Bluetooth smartphones every 30 seconds. Compared with Wi-Fi and cellular location, they show that Bluetooth can provide an order of magnitude more precise proximity detection. Montanari [42] proposes to use Bluetooth Low Energy (BLE) to measure the duration and the proximity of social contact using BLE-enabled wearables. It has been also used to measure, understand, and predict how individuals change their social behavior in response to infectious diseases [7]. Yoneki [7] uses Bluetooth to collect proximity devices data to measure, understand, and predict how individuals change their social behavior in response to infectious disease. Jamil *et al*. [43] use BLE tags and smartphones to track group dynamics in a massive religious gathering. It investigates the best configurations for the BLE tags and the scan durations for smartphones. Compared with the infection study, the group dynamics study requires detection in farther distances at more than 10 m. Also, the tags unilaterally advertise, and the smartphones unilaterally scan. It also does 10% duty cycling, with 5 minutes of hibernation between 30 seconds scans. Therefore, this does not fit with the continuous monitoring need for infectious contacts that could happen any time. A recent study also points out the inefficiency of the Bluetooth (LE) protocol in connection-based interactions when there are hundreds or even thousands of BLE devices in the communication range of each other [44]. Harris *et al*. [44] consider the dense BLE deployment scenario where hundreds or even thousands of tags interact with a large number of scanning devices such as smartphones. It raises the message collision and consequent energy waste issues of the BLE active scanning mode, and proposes an optimization scheme to solve them. Although Bluetooth technology has many desirable properties, it relies on the beacon exchange to detect each other. The beacons can reveal the identity of the transmitting device, threatening the privacy of the user.

### F. Cellular Network

Communication traces obtained by mobile phones are known to be good proxies for the physical interaction network, and they may provide a valuable tool for contact tracing. For example, calls and messaging activities were used to construct human contact networks [45]. Mobile network data or call detail records (CDRs) have also been used to model population flows, major mobility hubs, and movement typologies, and how they change as the Ebola outbreak unfolds [46]. We could even use two phones attaching to an identical cell as a signal for a possible physical contact. However, the coverage of a single cell tower is at least a few hundred meters in radius, so it would be too coarse to identify infectious physical contact events within a few meters [18].

### G. Accelerometer

One important instance of the mix encounters with strangers is public transport such as train or bus, which people can share for long enough time to enable several modes of disease transmission. For example, an infected person openly coughing in the bus can infect fellow riders in case of aerosol or droplet transmission diseases. When there are many potential contacts that a confirmed case cannot identify or recollect as in public transports, a potent tool we can exploit is the mobile devices such as smartphones. These devices can be leveraged to detect co-location, which can be a good proxy for the physical contacts. For instance, two smartphones located in adjacent cars in a train, both close to the doors dividing the cars, will probably exhibit high similarity in all their measures. But on a multi-car vehicle, a more relevant question in the context of epidemic infection is whether two passengers are on the same car or on different cars. So, in this letter, we explore how we can differentiate locations in the same train at car-level granularity. Our study reveals that accelerometer readings during train stop and start events tend to be characteristic of different car positions, so they can be used to generate a strong co-location signature on the car level. Thanks to the movements of the train, it does not require a complex communication infrastructure on the train for classification [41], but an accelerometer.

### H. Ambient Sound Sensor

Common ambient sound detection using the microphone sensor [47]–[49] can be a technology of choice. But using the microphone sensor has its own issues. First, the number of samples at its typically high sampling frequencies (*e.g.* 44.1 KHz) is too large for continuous and indefinitely long monitoring required for detecting contacts that can happen at any time. Second, privacy can be violated because any conversations are also recorded. Finally, there is the possibility of false detection. For example, two people watching the same TV channel or listening to other broadcast sounds in different places can be classified as coexistent.

### I. Magnetometer

The smartphone magnetometer has been extensively used for indoor localization and tracking (but not much for coexistence detection). Researchers found that the indoor magnetic field is rich in spatial features [16], and easy to sense [14]. Moreover, the field is stable over long periods of time [50]–[52]. The richness and the stability of the magnetic field enables mapping (a.k.a. fingerprinting) and magnetic map-based applications. The first application is indoor location. Chung *et al.* [15] showed that the geomagnetic anomaly can provide signatures for indoor locations that can be leveraged for sub-meter-level location accuracy. Frassl *et al.* [14] used magnetic maps with centimeter-level accuracy to localize a human or robot. Li *et al.* [52] discussed possible issues that can affect the precision and the feasibility of the fingerprinting approach for indoor location. Angermann *et al.* [50] found that the use of all three field components provides good resolution of ambiguities in a small indoor area. Carrillo *et al.* [53] used the three components of the measured magnetic field by smartphone magnetometers instead of just the intensity to improve accuracy. The second application is navigation. Brzozowski and Kazmierczak [54] discussed ways of recording, visualizing, and mapping local magnetic field changes in 3D that can be used as a support for indoor navigation systems for unmanned aerial vehicles (UAVs). Riehle *et al.* [55] considered a leader-follower style navigation application for visually impaired people where there is time gap between traversals, without relying on expensive indoor magnetic fingerprinting. A follower could compare its own magnetometer trace and the leader’s to determine if the follower reached a waypoint and if the follower went off-route. The third application also does not require fingerprinting, and it is of our interest in this paper – coexistence detection. Nguyen *et al.* [13] used only smartphone magnetometers to detect co-location of passengers in public transport. They exploited the fact that the passengers share the trajectory between at least two consecutive stations, and the magnetometer traces exhibit high similarity, which was measured by the distance in Derivative Dynamic Time Warping (DDTW). Kuk *et al.* [11] showed that even in outdoors the magnetometer traces can be compared to detect contacts within a few meters where the current GPS can have an order-of-magnitude larger errors. It showed that two closely located smartphones generate highly correlated magnetometer traces, which can be exploited to detect coexistence. Kuk *et al.* showed that they could lower the frequency to 1 Hz without significantly harming the detection performance, but increasing the battery life significantly.

The smartphone magnetometer overcomes undesirable properties of other technological alternatives. It can detect contacts within very short distances that fit infectious disease transmissions monitoring, and it can work indoors. It is supported by all smartphones, and works without any infrastructure support. It consumes relatively small power compared with other sensors, and has little privacy concerns. In this paper, therefore, we focus on the contact detection on smartphone magnetometers and explore their potential to provide a diagnostic tool for potentially infectious contacts made between smartphone holders.

As to the privacy concern of some of the technologies above, it may not an issue in the event of an epidemic. Authorities may legally have purpose-based access to the phone data of the infected or so suspected person, or rather, users may voluntarily give consent to the authority to use their trajectory data. Indeed, we assume such model in subsequent discussions.

Finally, it is worthwhile to mention that any combination of the magnetometer-based method proposed in this paper with other technologies is possible. For instance, the cost of comparing two traces for checking close contacts could be avoided if their GPS coordinates or cellular attachments show totally different values. Many valuable combinations could be conceived, but in this paper, we focus on the smartphone magnetometer-based method first so that it can be used in such combination in a more intelligent way.

## III. A Magnetometer-Based Diagnostic Test

**FIGURE 3.**
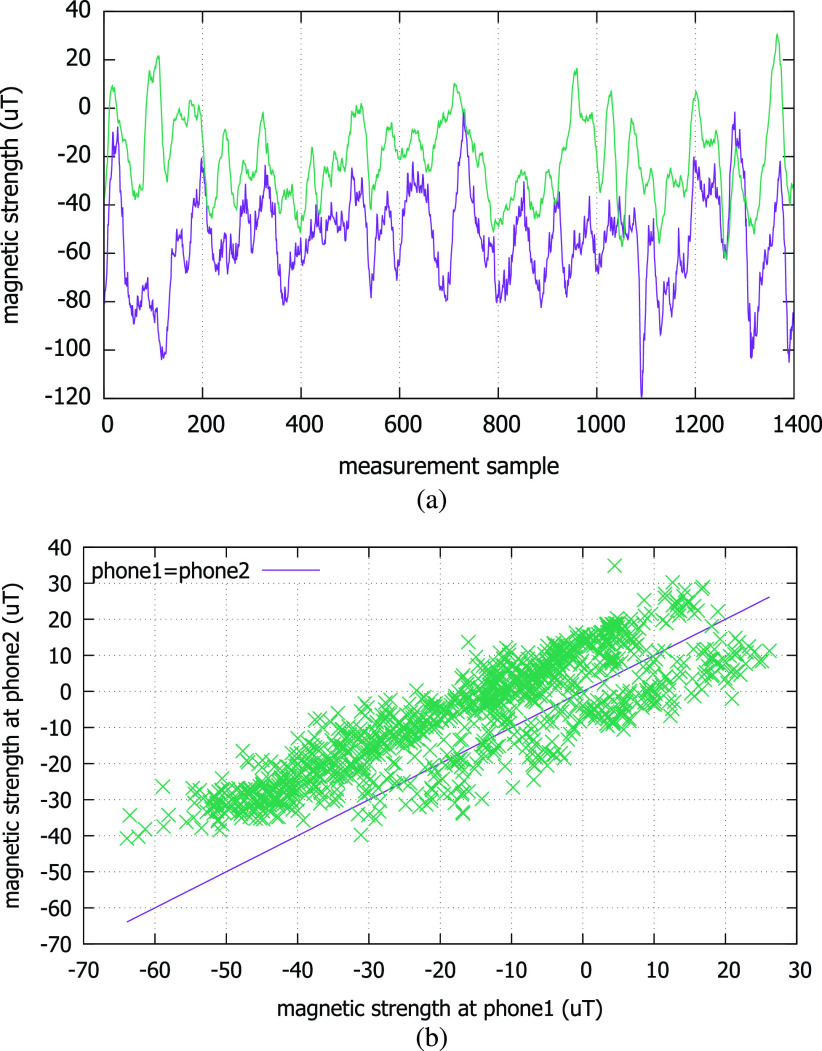
The magnetic field strength traces from two phones carried in proximity shows a linear correlation. (a) Two smartphone magnetometer traces from a co-existing context. (b) Linear correlation.

In this section, we discuss how we measure the similarity of two magnetometer traces. Among many similarity measures we can use, we pick the Pearson correlation coefficient. It is a good measure of linear correlation, which fits the linear correlation that two magnetometers in close proximity show in their ambient magnetic field strength readings. Fig. 3 shows real traces generated by two phones held by the people walking side-by-side through a corridor in a university campus building. Here, we let the phones measure the ambient magnetic field strength in $\mu \text{T}$ at the rate of 10 Hz. In (a), the horizontal axis is the sample number of measured magnetometer values, and the vertical axis is the strength of the magnetic field vector perpendicular to the ground. We observe that these two time series do exhibit similar fluctuations. The fluctuations are the results of the magnetic distortions to the geomagnetic field by ferromagnetic materials such as steel doors, pillars, and rebars among others in the building the smartphone users are passing by. The synchronized fluctuations of the two magnetometer readings have a linear correlation, as shown by (b). Therefore, when each phone records such trace while the user moves around in daily life, we can let the users or the disease control authority later check for possible contacts with an infected person using the strength of the linear correlation. For example, as in Fig. 1, a susceptible user can check if her smartphone has a trace segment that computes a high correlation with an infected person’s trace that can be provided by the disease control authority.

### A. Similarity Measure: Pearson’s R

In order to compute the similarity of two smartphone magnetometer traces, we need to use a similarity measure. There are numerous similarity measures, but some popular ones in the literature are cosine similarity, Dynamic Time Warping (DTW) distance, Euclidean distance, Kullback-Liebler distance, Jaccard similarity, Pearson correlation, among others [56]. In the areas of epidemiology and psychology, however, the measure of association is frequently analyzed by correlation analysis and regression analysis [57]. In this paper, we use the correlation analysis. As for the correlation measures, there are Pearson, Kendall, and Spearman correlation coefficients [58]. Among these, we pick the Pearson correlation coefficient, as it is a good measure for a linear correlation. To start with, Table 2 lists the notations we use in the subsequent discussion on how we compute the Pearson’s correlation coefficient of two magnetometer traces.

**TABLE 2:** Notations Used in Trace Similarity Comparison

Notation	Meaning	(Default) Value
$L$	No. of samples in an entire compared trace	$l_{tx}\cdot f_{s}$
$L_{c}$	No. of samples generated during an actual contact	$T_{c} \cdot f_{s}$
$l_{tx}$	Time window of interest (e.g. disease transmissible duration; see Fig. 2)	Disease-specific
$T_{W}$	Time length of the comparison window	[10, 120] sec.
$T_{c}$	Actual contact duration ($=l_{2}$; see Fig. 2)	–
$N_{W}$	No. of samples in the comparison window $T_{W}$	$T_{W} \cdot f_{s}$
$f_{s}$	Magnetometer sampling rate used in trace	10 Hz
$\theta _{c}$	Threshold for contact decision	[0.1, 0.9]
$A_{k},B_{k}$	$k$’th sample in the traces A and B, resp.	–
$\mu _{A}, \mu _{B}$	Means of samples in the traces A and B in $T_{W}$, resp.	–
$\sigma _{A}, \sigma _{B}$	Std. dev. of samples in the traces A and B in $T_{W}$, resp.	–
$\rho _{k}$	Correlation coefficient of $N_{W}$ samples from the traces A and B starting from $k$’th sample	–

**FIGURE 4.**
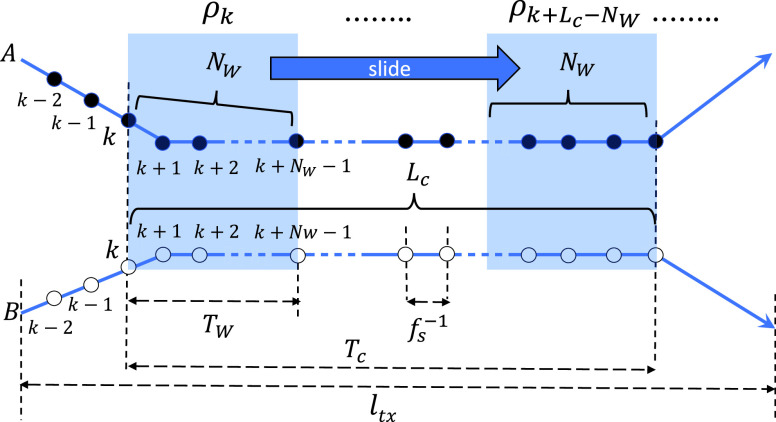
Notations used in the discussion.

Fig. 4 shows the relations between some of the notations. As two synchronized traces from the smartphones carried by two strangers are compared, we do not know whether or when the contact was made. As we discussed in Section I, we need to inspect the traces in a window of time $T_{W}$, as we slide the inspection window (blue box in Fig. 4) over the entire span of the traces that we are interested in ($l_{tx}$). Given $T_{W}$ and the magnetometer sampling rate $f_{s}$, the Pearson correlation coefficient for the samples in the window^1^ $N_{W}=T_{W}\cdot f_{s}$ starting from the $k^{\mathrm{ th}}$ sample is defined to be:

\begin{align*} \rho _{k}(A,B)=\frac {1}{N_{W}-1} \sum _{i=0}^{N_{W}-1} \left ({\frac {A_{k+i} - \mu _{A}}{\sigma _{A}} }\right) \left ({\frac {B_{k+i} - \mu _{B}}{\sigma _{B}} }\right) \\\tag{1}\end{align*}

where $A_{k+i}$ and $B_{k+i}$ are $(k+i)^{\mathrm{ th}}$ individual magnetometer readings from phones A and B, respectively. $\mu $ and $\sigma $ are the mean and the standard deviation of the measured values in two phone’s compared traces in the inspection window. Recollect that the existing works [11], [13] compute the similarity over the entire span of samples $L=l_{tx}\cdot f_{s}$, essentially making $N_{W} = L$. Unfortunately, at an arbitrary length $L$, we cannot control the false detection possibilities at all, whether positive or negative. Therefore, we will use a window $N_{W} \ll L$ to slide over the compared traces to find any interval for which $\rho _{k} (A,B)> \theta _{c},\,\,0 \le k < L-N_{W} + 1$, where $\theta _{c}$ is the cutoff threshold for the contact decision.^1^Since we use a constant value of $f_{s}=10$ Hz throughout the paper, we will use the term “window” to mean $N_{W}$ (which is equivalent to $10 \cdot T_{W}$) in subsequent discussions.

When $l_{tx}$ significantly increases, there are two aspects we need to consider: memory to store a trace (at smartphones) and correlation computation (at contact tracing check server). First, in terms of memory, the smartphones should keep the samples collected during the long transmissible duration. In our implementation, each magnetometer measurement sample is a vector, whose size is 50 bytes. At 10 Hz sampling, we generate data at 500 bytes/second. For an hour of continuous recording, it is approximately 1.8 MB. For one week, it is approximately 300 MB. Modern smartphones typically have a few tens of gigabytes of memory, so it will not be an excessive burden, especially in the emergency situation (*i.e.*, infectious epidemic). In terms of computation, the trace comparison is performed not on the smartphones but on a server to which the traces are submitted by users who want to check if they were in a close distance with the infected person. The correlation computation will take proportionally long to the length of the compared traces. But the computation itself is not extremely heavy. We tested the correlation computation with the sliding inspection window of 10 seconds over two continuous traces of 56,600 samples collected at 10 Hz (or $l_{tx} = 5,660$ seconds or 94 minutes). It takes approximately 6.7 seconds on a server that has an i7-7700K processor with the clock speed of 4.2 GHz, using only a single core. For week-long traces, it will be slightly over 10 minutes.

Note that the type of contact we aim to detect in this paper is coexistence [23] that will enable the ‘same-place-same-time’ (SPST) disease transmission. This contact type is more common in infectious disease transmissions than the ‘same-place-different-time’ (SPDT) type [59]. Since the smartphone users are assumed to stay/move together in this type of contact, we do not need to align the traces for the time gap and the moving speed differences by using such schemes as Dynamic Time Warping (DTW) [60]. Finally, we focus on the contacts made in the indoor contexts, because urban life is 90% indoors [61], and indoors is where most infection events take place.

### B. Parameters for Contact Decision

As the length of the traces $L$ over which the search is performed should be defined by the given disease of concern, *e.g.* by its incubation period [62] or the duration of active transmission, we do not consider this parameter further in this paper. As for the window size $N_{W}$, it should be long enough to find the contacts of the critical duration that can enable the transmission. However, it is hard to definitely characterize the duration as it will be disease-specific. So, in this paper, we focus on the technical side. Namely, we investigate the minimum window size that we can effectively use for the comparison, which will be equivalent to defining the granularity of inspection that smartphone magnetometers can offer. Longer contacts than the window size will manifest as a series of consecutive or densely grouped positive decisions, as we slide the window over the entire trace. Finally, we will show that the decision cutoff threshold $\theta _{c}$ is related with the window size $N_{W}$ for a given target detection accuracy.

If the magnetic field strength had a stationary distribution, we could easily draw earlier works on the sample size planning for clinical research [58]. Specifically, the required sample size $N_{W}$ over which the correlation is computed can be estimated as a function of the targeted cutoff $\theta _{c}$. In particular, $N_{W}$ decreases as $\theta _{c}$ or the confidence interval increases. Unfortunately, the distribution of the magnetic field strength measured by a moving smartphone is not stationary [13]. Without the stationarity of the magnetometer values in our environment, we cannot analytically derive the window size but turn to the measurement-based approach to estimate $N_{W}$ to meet the given $\theta _{c}$.

### C. Performance Measures

In order to see whether we can use the similarity check of the smartphone magnetometer traces as a diagnostic test, we evaluate its discriminative and predictive power. In particular, we need to evaluate it under different choices of $N_{W}$ and $\theta _{c}$. In clinical studies, numerous metrics are used to evaluate the quality of a diagnostic test. Some of them are: sensitivity, specificity, accuracy, positive and negative likelihood ratio, positive and negative predictive value, odds ratio, relative risk, risk difference, number needed to treat, *etc*. Among these, we will use the ones that are not affected by the prevalence, which can only be artificial in our setting.

**FIGURE 5.**
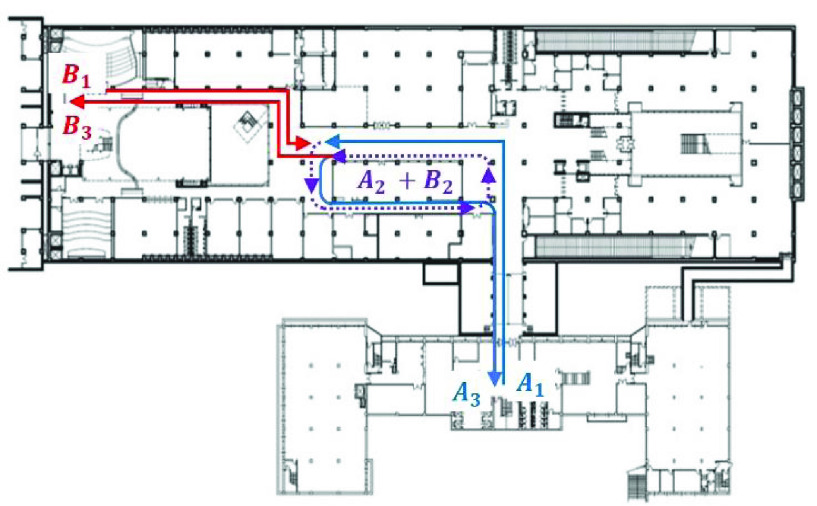
Two people meet and walk together for $T_{c}=90$ seconds before they part in an indoor space, making $L_{c}=900$ at $f_{s}=10$ Hz.

**FIGURE 6.**
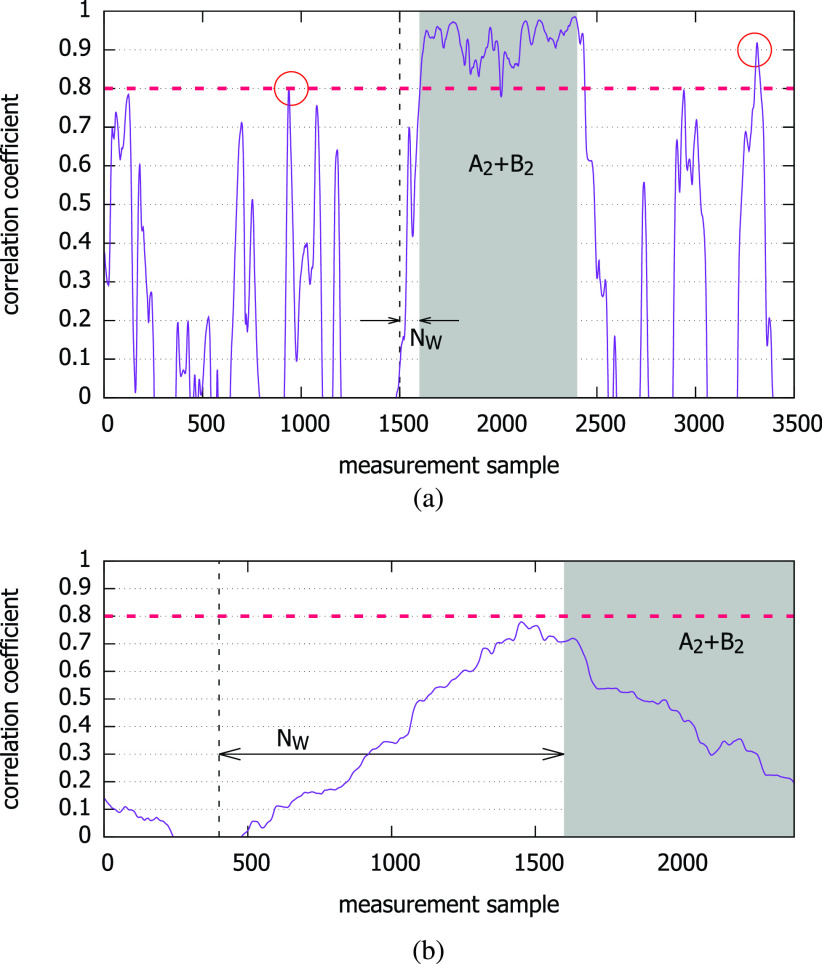
Decision changes with different window sizes (negative values not shown); shared regions represent the actual contact. (a) $N_{W}=100$. (b) $N_{W}=1,200$.

Given the ground truth (contact *vs*. no contact) and the decision using the smartphone magnetometer traces, there can be four cases among which true positive (TP) and true negative (TN) are desirable, and false positive (FP) and false negative (FN) should be minimized (Table 3). As in any other accuracy assessment of diagnostic tests, we use the $2\times 2$ table. As to how the false detections (FP and FN) arise in our setting, we can consider two possibilities. Suppose the length of the contact duration represented in the traces is $T_{c}$, and the number of samples generated during the duration $L_{c}=T_{c}\cdot f_{s}$. Then, let us consider Fig. 5, where two people move indoors with the smartphone magnetometers measuring the ambient field strength at 10 Hz. The two people come from different places ($A_{1} vs. B_{1}$), meet in the middle, and move together in the region labeled “$A_{2} + B_{2}$” for $T_{c}=90$ seconds, and then part and return to their initial locations ($A_{3} vs. B_{3}$). Fig. 6 shows the correlation coefficients obtained as we slide the inspection window over the entire trace under two different $N_{W}$ values. The x-axis is the sample number $k$ in (1) at which the coefficient is computed, and the y-axis is $\rho _{k}$. The shaded region represents the duration of contact. It is approximately from samples 1,600 through 2,400 in both graphs.

**TABLE 3:** Classification is a Function of $\theta_{c}$, Under a Given $N_{W}$

Test outcome	Ground truth condition		subtotal
	Contact	No contact	
$\rho _{k} > \theta _{c}$	TP	FP	TP+FP
$\rho _{k} < \theta _{c}$	FN	TN	FN+TN
subtotal	TP+FN	FP+TN	TP+FP+FN+TN

#### 1) Comparison Window is Narrower Than the Contact Duration ($N_{W} < L_{C}$)

**FIGURE 7.**
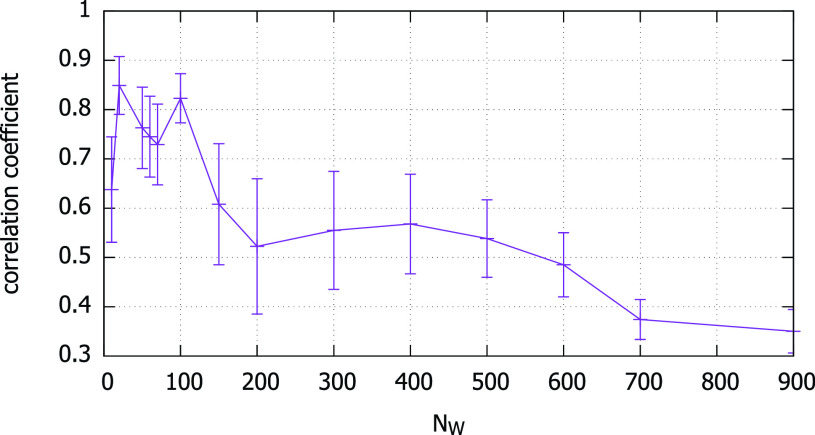
Pearson correlation coefficient $\rho _{k}$ with 95% confidence interval, for a large number coexistent trace pairs.

There are two subcases in this case. First, if $N_{W}$ is very small, it can cause many spurious contact detections since coincidental high correlations may not be sufficiently averaged out. For example, with $N_{W} = 100$ and 1,200, Fig. 6(a) and (b) show their Pearson correlation coefficients, respectively. The circles in Fig. 6(a) show that two spurious detection events are possible for $N_{W}=100$ and $\theta _{c}=0.8$. Second, even if $N_{W}$ is large there are still chances for false detections, but only negative. It is because increasing $N_{W}$ decreases $\rho _{k} (A,B)$ as a consequence of the non-stationarity of the magnetic field strength distribution [13], when the human smartphone holder moves through space. Using our coexistent trace pairs, we indeed confirm that the larger window sizes significantly reduce the correlation coefficient (Fig. 7).

#### 2) Comparison Window is Wider Than the Contact Duration ($N_{W} > L_{C}$)

In this case, a possible consequence is that the adjacent measurement samples outside the coexistence duration that happen to be included in the window decreases the cross correlation, possibly leading to a false negative decision depending on $\theta _{c}$. Observe that for high cutoff thresholds such as $\theta _{c}>0.8$, Fig. 6(b) will falsely determine that there was no contact, whereas the former will correctly detect the contact.

Either way, these problems can lead to false decisions about the contact, so it is clear that we need to determine the appropriate window size $N_{W}$ as well as the cutoff threshold $\theta _{c}$.

## IV. Evaluation

For our measurement-based study, we use indoor magnetometer traces collected in the Korea University campus in Seoul, Korea. Below, we first discuss how we collect the traces. Then we evaluate the smartphone magnetometer-based contact detection using the measures mentioned in Section III-C.

### A. Magnetometer Trace Collection

To collect the magnetometer traces, we developed and installed a magnetometer sensing app for Android smartphones, Samsung Galaxy S5, S6, S8 and LG G3 and G4. We confirmed that our app works correctly on all these platforms. Among the phones, we used two Galaxy S5’s to collect the traces used in this section. We synchronized their sensing activity through the Network Time Protocol (NTP) [63] for later comparison of their magnetometer traces. We collect the magnetometer traces in five different buildings in the campus. We picked three places in each building. At each place, we repeated the trace collection six times along the same walking path. So, in total, there are 90 traces, and each trace is 300 seconds long. There are $C(6,2)=15$ pairs of traces per place to be judged co-existent. Since there are 15 different places from which the traces were collected, we have $15\cdot C(6,2)=225$ co-existent trace pairs in total. On the other hand, there are $C(15,2)\cdot C(6,1)\cdot C(6,1)=15\cdot 7\cdot 6\cdot 6=3,780$ non-coexistent pairs.

We measured the magnetic field strengths at the default sampling frequency of 10 Hz, a popular magnetometer sensing rate in the literature [17]. The magnetometer readings are obtained in three phone-specific axes: X, Y, and Z. In order to simulate typical indoor walking dynamics, we let the smartphone holders walk approximately at the ‘preferred’ walking speed [64]. It is known that people prefer to walk at approximately 1.4 m/s (or 5.0 km/h) irrespective of cultures, as they find slower or faster speed uncomfortable. Each trace was produced in narrow corridors, and we saw to it that the traces do not deviate from each other more than an ‘arm’s length’ to simulate the typical personal gap [65].

As the smartphones can have arbitrary attitudes when and while the contact is made, the measured magnetic strengths in their X, Y, and Z axes will generally be misaligned. For comparison, therefore, they should be translated to a common coordinate system. For this, we use Android getRotationMatrix() method to translate the phone-specific coordinates to the absolute coordinate (*i.e.*, North, East, *etc*). A desirable property of the geomagnetism is that it has absolute reference directions such as the East and the North. Smartphones will change attitudes freely, but the translation method lets us readily compare the traces from different phones regardless of their attitudes. As to the robustness of the method against the accumulation of errors over a long duration of continuous operation, it is a research issue of its own [66]. In this paper, we assume that such calibration is being done to maintain the precision of the magnetometers.

**FIGURE 8.**
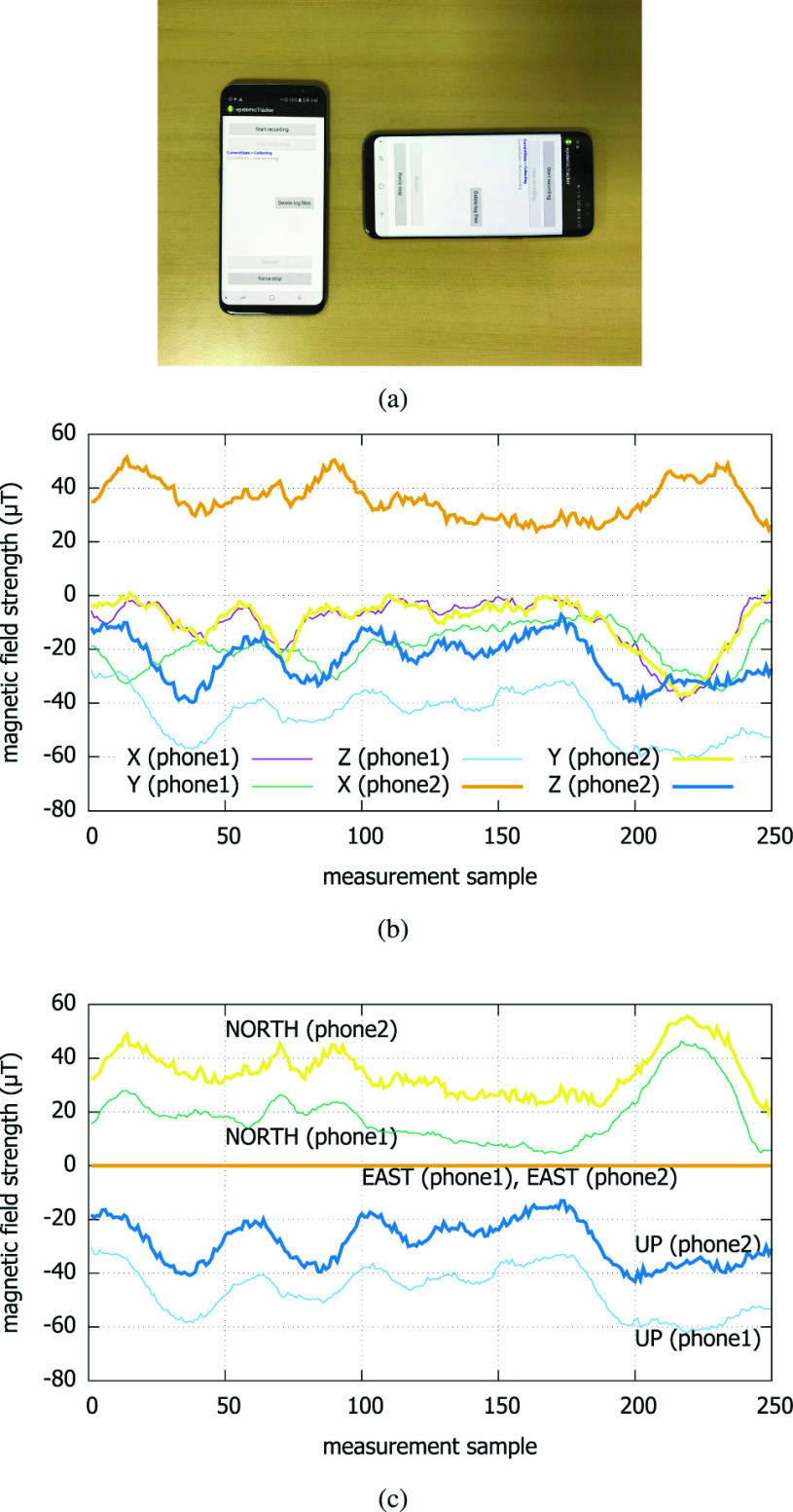
Two devices in arbitrarily misaligned attitudes translated by getRotationMatrix method. (a) Freely aligned phone attitudes. (b) Before translation. (c) After translation.

Fig. 8 illustrates the alignment operation in our measurement system. Under the misalignment (a), it is not straightforward to choose the axis for the comparison (b). The traces in (b) shows that the X-axis of Phone 1 is aligned with the Y-axis of Phone 2, which is the ground truth as shown in (a). But after the translation, the readings from the two coexistent but misaligned phones are cleanly separated along the three absolute axes (c). We notice that the Z-axis traces from (b) are identical to UP-axis traces in (c), because the phones were held parallel to the ground (a) in the generation of the traces in (b). Finally, the EAST is simply the cross product of the two vectors NORTH and UP, so it is redundant. Thus in our implementation, we choose whichever axis between NORTH and UP that shows the highest correlation in the decision.

### B. Results

Here, we compute the evaluation measures for the combinations of the window size and the decision threshold. In particular, we will compute them for the first $N_{W}$ samples from each trace pair, *i.e.*, $k=0$ in (1). But first, there is a caveat.

#### 1) On Prevalence and its Dependent Measures

In total, there are 3, 780 and 225 non-coexistent and co-existent trace pairs in our data set, totaling at 4, 005. The Prevalence in our data set is thus 225/4,005 = 5.62 %. However, this is artificial – we could have made it higher or lower by producing more of coexistent or non-coexistent traces, respectively. Naturally, it is meaningless to calculate the measures affected by the prevalence, where the prevalence of disease is artificially controlled [22]. Sensitivity and specificity are not generally related to the prevalence of the disease in the population considered, since these are properties of the diagnostic tool. Unlike sensitivity and specificity, measures such as predictive values, accuracy, relative risk and risk difference are affected by the prevalence. Therefore, we exclude them, and use the measures that are not affected by the prevalence to evaluate the magnetometer-based contact test.

#### 2) Sensitivity and Specificity

Sensitivity is expressed as the proportion of correctly classified as true positives among the total contacts $TP/(TP+FN)$. In other words, it is the ability of the magnetometer-based test to correctly identify the trace pairs with a real contact. A highly sensitive test is useful, when we do not want to miss a contact (with an infected person) in screening the population. The specificity is the ability to identify the no contacts, expressed as $TN/(TN+FP)$. A specific test will rarely misclassify the trace pairs without a contact as having made a contact. The sensitivity and specificity show the discriminative powers of a diagnostic test.

**FIGURE 9.**
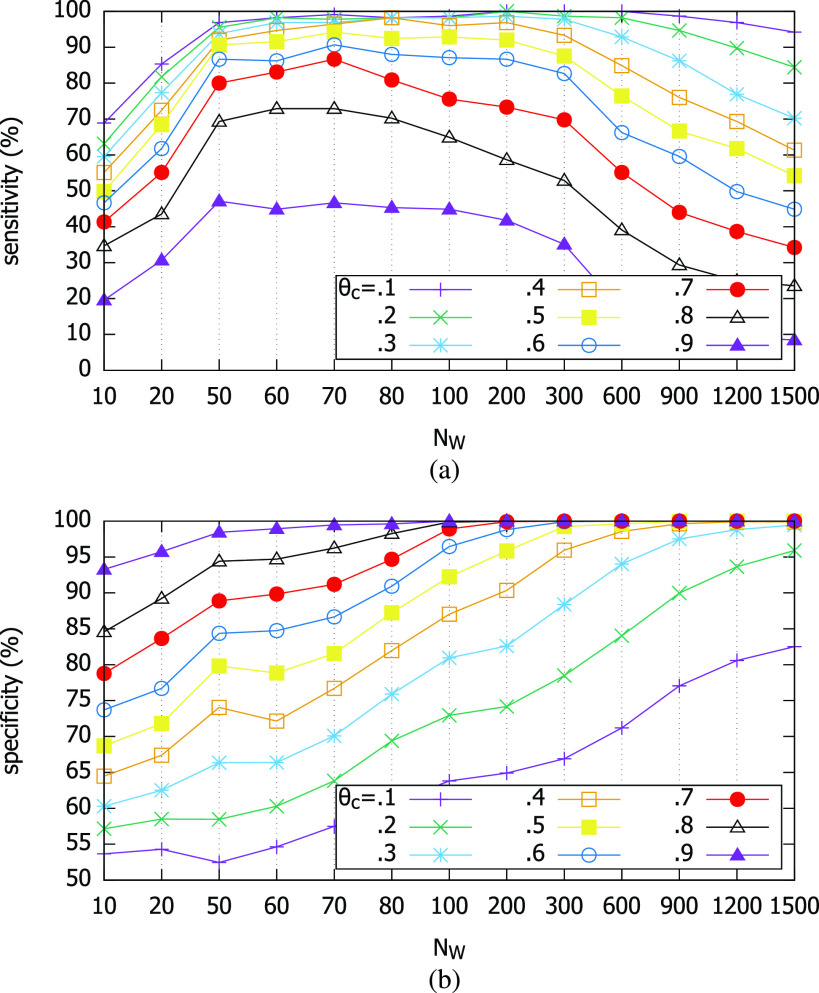
Sensitivity and specificity of the magnetometer-based contact test. (a) Sensitivity. (b) Specificity.

Fig. 9 shows the sensitivity and the specificity of our smartphone magnetometer-based test, as functions of $N_{W}$ and $\theta _{c}$. We first find that larger $N_{W}$ does not necessarily mean the higher sensitivity. Although happening at different values of $N_{W}$ ($50|_{\theta _{c}=0.9}\,\,\sim \,\,200|_{\theta _{c}=0.1}$), the sensitivity begins to decrease beyond a certain $N_{W}$ at each cutoff threshold. It implies that the correlation decreases when computed for an excessively long trace segment used as the inspection window. This is due to the non-stationary property of the magnetometer measurement value distribution [13]. The specificity, on the other hand, steadily increases as we use larger $N_{W}$. The lesson here is that when we use the magnetometer-based diagnostic test, we need to examine the similarity of the two traces using the time window of $N_{W}=50 \sim 200$ to achieve the highest sensitivity. Then, the choice of the exact cutoff threshold will depend on the target specificity.

**FIGURE 10.**
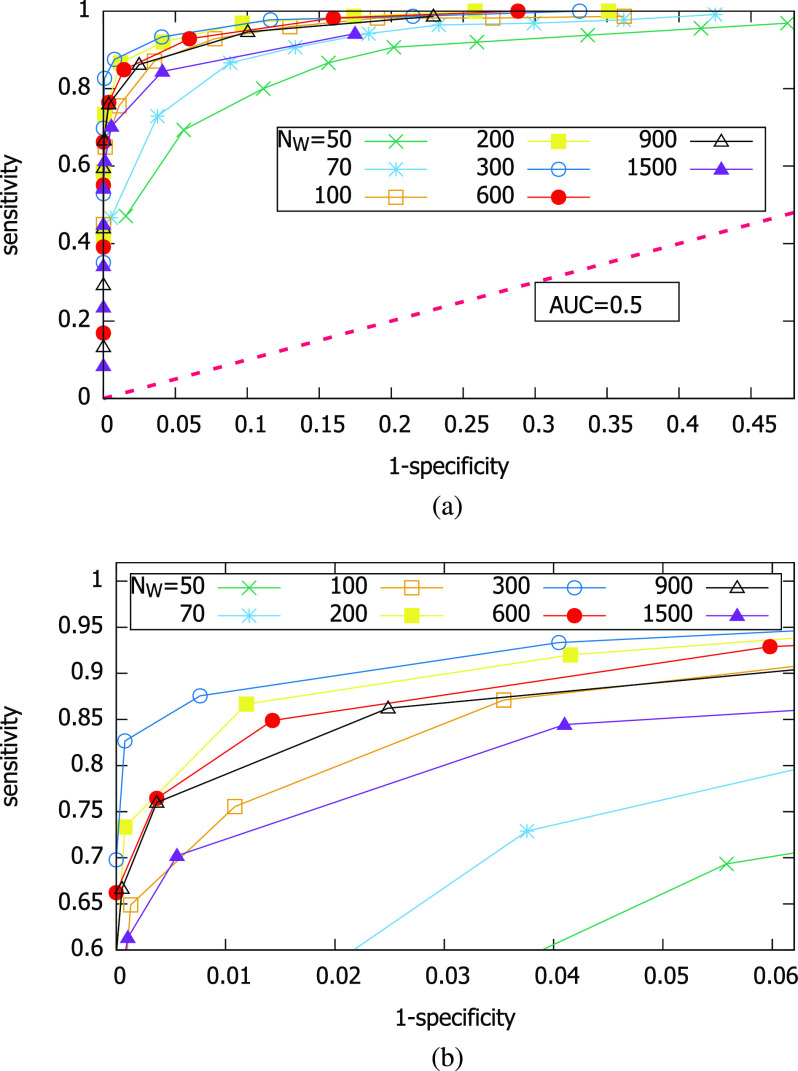
ROC curves obtained by varying $\theta _{c}$ for different values of $N_{W}$. (a) ROC. (b) ROC.

Also, we find in Fig. 9 that the sensitivity is higher with lower cutoff values, whereas the specificity is higher with higher cutoff values. This tension is natural, and can be summarized in the Receiver Operating Characteristic (ROC) curve. Fig. 10 shows the ROC curves for different parameter combinations. Although we cannot show the area under curve (AUC) itself due to the absence of very low specificity data points, it is clear that the AUC’s for various $N_{W}$ are very high. Namely, the magnetometer-based test is of high diagnostic quality. Among the inspection sample window sizes, very small $N_{W}$ (50, 70) and very large $N_{W}$ (1,500) lead to poorer AUC than those in the middle ($N_{W}= 100 \sim 900$) as shown in Fig. 10(b). $N_{W}=300$ achieves the best overall AUC.

In order to obtain the cutoff value $\theta _{c}$ that achieves the highest AUC for a given $N_{W}$, we can compute the shortest distance from (0, 1) to the curves. Table 4 shows the result. This table shows what $N_{W}$ to use in case a cutoff threshold is given. It is worthwhile to notice that for higher cutoff thresholds, the window size does not have to be large. In particular, $N_{W}$ can be less than 100 for very high cutoff thresholds. The table can serve as a guideline to choose appropriate values for the parameter pair.

**TABLE 4:** $\theta_{c}$ That Leads to the Shortest Distance From (0,1) to the ROC Curve for Different $N_{W}$

	$\theta _{c}$	$N_{w}$
Lower cutoffs	0.1	1,500
	0.2	900
	0.3	600
	0.4	300
	0.5	200
Higher cutoffs	0.6	150
	0.7	70
	0.8	70
	0.9	50

#### 3) Likelihood Ratios

Likelihood ratio (LR) is the mostly widely applied measure of diagnostic accuracy. Also, it can serve as a predictive measure. In our context, LR tells us how many times more likely a decision is in the trace pairs with the contact than in those without contact. When both probabilities are equal (*i.e.*, $LR=1$), such test is of no value. The LR for positive test results ($LR+$) is defined as $\frac {TP}{TP+FN} / \frac {FP}{TN+FP}$. The higher the $LR+$, the more indicative the test is of the contact. Good diagnostic tests have $LR+ > 10$ and their positive result has a significant contribution to the diagnosis [67]. On the other hand, the LR for negative test result ($LR-$) is defined as $\frac {FN}{TP+FN} / \frac {TN}{TN+FP}$, and it represents the ratio of the probability that a negative result will occur in trace pairs with the contact to the probability that the same result will occur in trace pairs without the contact. Good diagnostic tests have $LR- < 0.1$ [67]. The lower the $LR-$, the more significant contribution of the test is in ruling-out. LR’s do not depend on prevalence of disease of population, as only sensitivity and specificity values are used to calculate them. As a result the LR’s of one study could be used in another setting with the condition that the definition of contact is not changed.

**FIGURE 11.**
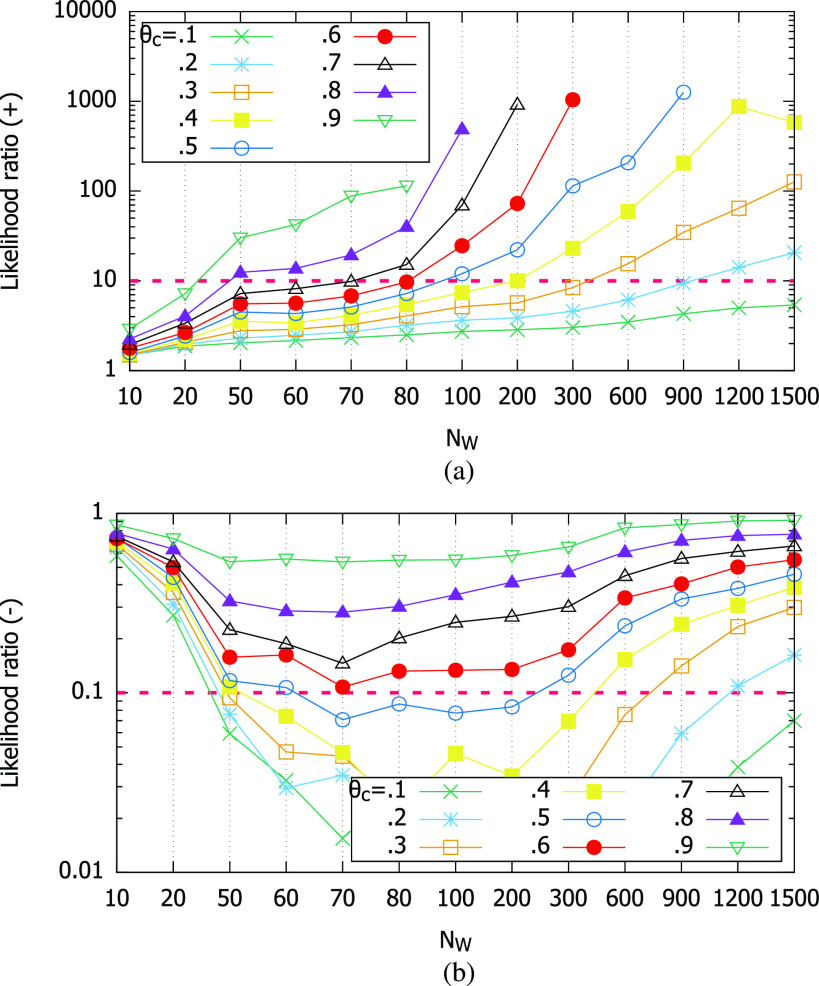
Likelihood ratios. (a) Positive likelihood ratio. (b) Negative likelihood ratio.

The likelihood ratios of the smartphone magnetometer-based contact test are shown in Fig. 11. In (a), we observe that it is highly useful for positive identification of contacts. The criterion $LR+ > 10$ tells us that the positive likelihoods can be a significant contribution to the diagnosis. We also note that we do not need large $N_{W}$ to have $LR+ > 10$, especially when we use higher cutoff thresholds of $\theta _{c} \ge 0.6$. Less than 100 measurement samples at 10 Hz, or equivalently 10 seconds, is enough to qualify for a good test for positive identification of contacts. On the other hand, Fig. 11(b) shows that the higher cutoff thresholds cannot achieve $LR- < 0.1$ regardless of $N_{W}$. It implies that using the higher cutoffs can produce a high fraction of false negatives. However, this issue may be mitigated if we require that a contact duration be composed of a series of positive decisions as we slide the inspection window. For example, in Fig. 6(a), hundreds of adjacent positive decisions will occur as we slide up $k$ in (1). Interspersed false negatives will less affect the final decision then.

#### 4) Diagnostic Odds Ratio

Diagnostic odds ratio (DOR) is a relative measure for diagnostic accuracy, used for the estimation of discriminative power of diagnostic procedures [67]. DOR of a test is the ratio of the odds of positivity in traces with the contact relative to the odds in traces without contact. It is calculated according to the formula: $DOR = (TP/FN)/(FP/TN)$. DOR depends significantly on the sensitivity and specificity of a test. A test with high specificity and sensitivity with low rate of false positives and false negatives has high DOR. With the same sensitivity of the test, DOR increases with the increase of the test specificity. For example, a test with sensitivity > 90% and specificity of 99% has a DOR greater than 500. The diagnostic odds ratio ranges from zero to infinity, although for useful tests it is greater than one, and higher diagnostic odds ratios are indicative of better test performance.

**FIGURE 12.**
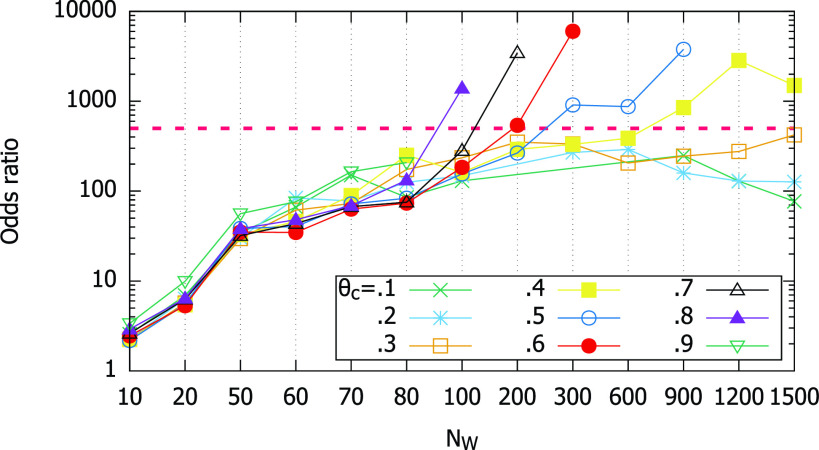
Odds ratio for different $\theta _{c}$ with 10 Hz sampling.

Fig. 12 shows that the DOR of the smartphone magnetometer-based contact test is much larger than one for most $N_{W}$ values. So, this measure also confirms that the magnetometer-based test is useful. If we use $OR=500$ as the example criterion, the figure tells us that higher cutoff thresholds qualify with less measurement samples $N_{W}$ to look at ($\theta _{c}=0.9$ has $FN=0$ at $N_{W}=100$, so it should qualify although we cannot plot it). For these higher cutoffs, less than 300 samples (or equivalently 30 seconds) or less is enough to achieve the high DOR.

### C. Summary of Results and Recommendations

Above, we evaluated the quality of the smartphone magnetometer traces comparison as a clinical test for potential (infectious) contact. All evaluation metrics that we used for the evaluation, namely sensitivity, specificity, likelihood ratio, and diagnostic odds ratio, point to the fact that the number of magnetometer readings to be compared between two traces ($N_{W}$) can be small. These metrics produce slightly different optimal numbers for the required readings, but if we need one good number to apply in real-life cases, it is 100 samples (or equivalently 10 seconds at the 10 Hz sampling frequency). It leads to the best or close-to-the-best performance in all the evaluation measures. Our recommendation is that when two magnetometer traces from two smartphones are compared, *one needs to use a window of 100 samples*^2^ *for the Pearson correlation computation to achieve the most precise decision* as to whether the contact was really made between the smartphone holders. One further recommendation is that the correlation coefficient value used as the decision threshold can be high. Specifically, $\theta _{c}=0.6$ is a good match for the 100-sample inspection window. Note that these two numbers $N_{W}$ and $\theta _{c}$ to produce the most precise decision are closely related, and other combinations than (100, 0.6) can be inferred from the results in the previous section.^2^At 10 Hz. For other sampling frequencies, it should be adjusted to the number of samples generated in 10 seconds.

## V. Conclusion

When a large-scale epidemic crisis unfolds in the highly urbanized society today, the traditional contact tracing method of medical personnel interviewing the infected persons will become highly costly, slow, and ineffective. In this paper, we discuss how smartphones carried by most people can be harnessed to automatize the contact tracing in such situation. We exploit the fact that smartphone magnetometers show high linear correlation when two phones coexist within a short, disease-contractible distances, such as less than two meters. Then, we use a battery of metrics to evaluate the value of such smartphone magnetometer traces comparison as a clinical test that medical personnel can use in real-life with a high trust level. Our evaluation reveals that the magnetometer-based method qualifies for a valid clinical test, if used with certain parameter values in the correlation computation. Specifically, our finding and recommendations are as follows. First, the size of the sliding window of trace section to be compared is best to be what corresponds to 10 seconds of samples. Second, the decision threshold that matches the comparison window size is 0.6, for the most precise contact decision. These two parameters are inversely related with respect to the precision of the contact detection, and other combinations around the recommended values are also possible. In future, we will further test the reliability of the proposed method with the recommended and other parameter settings in more extensive real-life environments, for instance with different smartphone movement speeds, with obstacles, people or objects between or around the smartphone holders, and with interferences such as power lines close to the smartphones. The artificial traces that we generated in a controlled environment could have biased our experiment results and our conclusion. Therefore, we will need to optimize the proposed method further against the real-life traces in a building or in public places to make it more reliable and actually usable in the real-life epidemic situations.
